# Cell Viability, Drug Screening, and Mechanism Study Under Mild Phototherapy Integrated Chemotherapy (PIC)

**DOI:** 10.1002/advs.202502836

**Published:** 2025-08-29

**Authors:** Tongge Li, Shihui Wang, Qianhui Ling, Junyi Sun, Ning Yang, Zhanglei Fu, Zhaoyuan Zhang, Si Chen, Yanfei Wang, Ni Yu, Yafei Wang, Zhexuan Ding, Haodong Liu, Yanwei Jia, Yifang Wang, Xingcai Zhang

**Affiliations:** ^1^ School of Electrical and Information Engineering Jiangsu University Zhenjiang 212000 China; ^2^ Department of Medicine Stanford University School of Medicine Stanford CA 94305 USA; ^3^ School of Bioengineering Chongqing University Chongqing 400030 China; ^4^ Fluid Machinery Center Jiangsu University Zhenjiang 212000 China; ^5^ School of Agricultural Engineering Jiangsu University Zhenjiang 212013 China; ^6^ State‐Key Laboratory of Analog and Mixed‐Signal VLSI Institute of Microelectronics Faculty of Science and Technology‐ECE University of Macau Macau 999078 China; ^7^ School of Innovative Design City University of Macau Macau 999078 China; ^8^ John A. Paulson School of Engineering and Applied Sciences Harvard University Cambridge MA 02138 USA; ^9^ World Tea Organization Cambridge MA 02139 USA; ^10^ Department of Materials Science and Engineering Stanford University Stanford CA 94305 USA; ^11^ School of Engineering University of California San Diego La Jolla CA 92093 USA

**Keywords:** cell viability, drug screening, impedance, less bonding (LALB) mechanism, light‐aiding, photochemical coupling, phototherapy integrated chemotherapy (PIC), visualization

## Abstract

Phototherapy integrated chemotherapy (PIC) offers a promising cancer treatment strategy as single chemotherapy approaches are less effective due to tumor heterogeneity and drug resistance, and phototherapy often involves harmful radiation, and the potential of mild phototherapy to enhance chemotherapy remains underexplored. Cancer cells, as key drivers of tumor metastasis, are crucial targets, but current methods for visualizing their response to theranostics are slow. Here, nondestructive real‐time impedance spectroscopy to monitor cancer cell response, identifying an optimal light exposure time of 60 min and defined cell viability and resistibility through impedance measurements, achieving high correlation (92.45% and 99.82%) with standard methods is used, and it is demonstrated that mild phototherapy significantly enhance chemotherapy, especially with Lenvatinib in liver cancer cell treatments. Through an in‐depth study, the “more light‐aiding, less bonding (LALB) mechanism,” meaning less conjugated chemical bonding within the excitation threshold, results in more pronounced light‐aiding properties, is proposed. This photochemical interaction mechanism confirms that combining phototherapy and chemotherapy offers significant benefits with higher targeting and lower toxic side effects in cancer and disease treatments, providing a new strategy of photochemical interaction and coupling for drug screening and mild phototherapy integrated chemotherapy (mild‐PIC) treatment evaluation, and further promotes the development of other advanced biomedical applications.

## Graphical abstract

Mild phototherapy integrated chemotherapy (mild‐PIC) monitored by real‐time impedance spectroscopy enhances drug efficacy in liver cancer via the “More Light‐Aiding, Less Bonding (LALB)” mechanism, achieving higher targeting with lower toxicity.

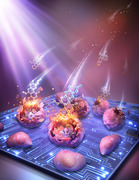

## 1 Introduction

Cancer metastasis and dissemination are the primary causes of treatment failure, with cellular heterogeneity and drug resistance posing significant challenges in current oncology.^[^ ^1^, ^2^ ^]^ Traditional monochemotherapy offers limited efficacy in addressing the complex and dynamic nature of cancer, not only threatening patient survival but also placing a substantial burden on healthcare systems.^[^ ^3^ ^]^ Therefore, identifying more effective therapeutic strategies is of considerable clinical and societal importance.^[^ ^4^, ^5^ ^]^ Among emerging approaches, the combination of chemotherapy and phototherapy has garnered increasing attention due to its potential synergistic effects, aiming to overcome the limitations of monotherapy.^[^ ^6^, ^7^ ^]^ To maximize the benefits of this combined treatment, precise methods for assessing therapeutic efficacy and visualizing cancer cell status are essential. Such tools enable the optimization of treatment duration and dosing in photochemotherapy.^[^ ^8^ ^]^ By advancing combination therapies and enabling real‐time visualization of cellular responses, cancer reatment toutcomes can be significantly improved. Moreover, this approach holds promise for facilitating the development of personalized medicine,^[^ ^9^ ^]^ while also driving innovation in related fields such as life sciences research, molecular breeding, and drug screening.^[^ ^10^ ^]^

Chemotherapy remains a widely used modality for cancer treatment.^[^ ^11^ ^]^ For instance, Bell et al. enhanced the transcription of cyclooxygenase‐2 (COX‐2) in cancer cells using chemotherapeutic agents to inhibit cancer cell proliferation.^[^ ^12^ ^]^ Zhan et al. identified the role of lysyl oxidase (LOX) in promoting mitochondrial ferroptosis during chemotherapy, effectively suppressing the progression of liver cancer.^[^ ^13^ ^]^ However, drug resistance continues to be a major obstacle in the treatment of most human cancers.^[^ ^14^ ^]^ Debaugnies et al. elucidated the mechanisms of key regulatory enzymes involved in chemotherapy resistance,^[^ ^15^ ^]^ while Low et al. demonstrated that dual‐specificity phosphatase 16 (DUSP16) can accelerate apoptosis and modulate drug resistance in various cancers.^[^ ^16^ ^]^ Pietilae et al. investigated how adaptive adhesion dynamics of cancer cells contribute to drug resistance and influence the invasive potential of ovarian cancer during chemotherapy.^[^ ^17^ ^]^ Although chemotherapy can exert certain inhibitory effects on cancer cell activity and metastasis under conditions of effective drug resistance control,^[^ ^18^ ^]^ research suggests that multimodal strategies may offer superior therapeutic outcomes. These include reducing drug resistance, lowering cancer recurrence rates, and minimizing damage to healthy tissues.^[^ ^19^ ^]^ Deng et al. reported that combining radiotherapy and chemotherapy under the guidance of specific inhibitors can significantly enhance treatment efficacy.^[^ ^20^ ^]^ Similarly, Au et al. developed nano‐conjugated agents capable of delivering cytotoxic chemotherapeutics, where radiotherapy was found to enhance their therapeutic performance, resulting in a more potent anticancer effect.^[^ ^21^ ^]^ In our own work, we developed microalgae‐based oral microcarriers aimed at maintaining gut microbiota homeostasis and protecting intestinal integrity during cancer radiotherapy.^[^ ^22^, ^23^, ^24^, ^25^ ^]^

Phototherapy is a noninvasive and minimally radiative adjuvant approach for cancer treatment,^[^ ^26^, ^27^, ^28^, ^29^ ^]^ offering advantages such as reduced systemic toxicity and targeted therapeutic effects.^[^ ^30^, ^31^, ^32^, ^33^, ^34^, ^35^, ^36^, ^37^, ^38^, ^39^, ^40^ ^]^ Currently, both photodynamic therapy and photothermal therapy have been applied in clinical oncology.^[^ ^41^ ^]^ In our study, we developed novel materials integrated with phototherapy and demonstrated their multimodal anti‐tumor efficacy against specific cancer cells both in vitro and in vivo.^[^ ^42^ ^]^

Monitoring the status of cancer cells is essential for evaluating the effectiveness of cancer therapies. Assessing cellular metabolic activity or structural alterations is key to determining cell viability.^[^ ^43^, ^44^, ^45^, ^46^ ^]^ Traditional invasive methods, such as Trypan Blue staining and the CCK‐8 assay, can cause significant damage to cells and are unsuitable for continuous monitoring.^[^ ^47^, ^48^, ^49^ ^]^ To address these limitations, more advanced techniques have been developed for evaluating cell viability, including deep‐learning‐assisted terahertz imaging, flow cytometry, and microimaging.^[^ ^47^, ^50^, ^51^ ^]^ However, the accuracy of flow cytometry is highly dependent on sample preparation, while microimaging is sensitive to cell distribution and exhibits reduced capability in detecting cell adhesion.

To overcome these limitations, several accurate and nondestructive evaluation methods have been proposed.^[^ ^47^, ^52^ ^]^ For example, Tan et al. utilized hyperspectral stimulated Raman scattering (hSRS) imaging within the hydrocarbon (CcH) window and applied sparsity‐driven hyperspectral image decomposition to reveal the unique metabolic signatures associated with chemotherapy resistance in certain cancers.^[^ ^53^ ^]^ Li et al. established a model correlating diffraction fingerprint spectra with cell states at the single‐cell level, offering a label‐free and noncontact method for evaluating cellular activity.^[^ ^43^ ^]^ However, spectral approaches often suffer from weak signal intensity and low stability, necessitating advanced and sensitive instrumentation.^[^ ^54^ ^]^ In addition, electrochemical methods such as field‐effect transistors (FETs), potentiometric sensors, and other biosensor technologies have been explored for assessing cell viability. These techniques monitor cellular status by detecting changes in cell morphology, gene expression, and redox activity,^[^ ^55^, ^56^, ^57^, ^58^, ^59^, ^60^, ^61^, ^62^ ^]^ thereby improving the stability and accuracy of cell state assessment. Nonetheless, conventional evaluation methods still face challenges, including limitations in sample processing and the lack of real‐time, continuous monitoring capabilities.

We want to develop a technology that can evaluate and characterize cell status in real time, without labels and noninvasively, which has high application value for cancer cell monitoring and evaluation of cancer treatment effects. Especially, we propose that phototherapy integrated chemotherapy (PIC) can offer a promising cancer treatment strategy and we want to explore in‐depth study to reveal the effects of PIC and its guiding mechanism in cancer and disease treatments, providing a new strategy of photochemical interaction and coupling for drug screening and mild phototherapy integrated chemotherapy (mild‐PIC) treatment evaluation, and further promotes the development of other advanced biomedical applications.

We proposed a method of photochemical synergistic induction of cancer cell apoptosis, combined with impedance dynamic sensing technology, to achieve real‐time visual monitoring and mechanism analysis of cancer treatment effects. Compared with monitoring a single cell response parameter, we developed a two‐parameter impedance model to simultaneously quantify cell viability and resistance, providing a brand‐new dimension for the dynamic assessment of the synergistic effect of photochemotherapy. By optimizing the detection frequency and the duration of light stimulation, a cell state assessment framework based on impedance spectroscopy was constructed, which can nondestructively identify the key Windows in the apoptosis process of cancer cells, namely the optimal chemotherapy dose and phototherapy time. Using Huh‐7 as the model cancer, glycyrrhizic acid (GA) as the model drug, and normal liver cells (WR‐68) and YPF as the control group for verification, the invasive limitations of the traditional methods of trypan blue or MTT were broken through, and the marker‐free dynamic differentiation of cancer cell states during the treatment process was achieved. Further clarify the structure‐activity relationship between the conjugation degree of drug molecules and the photochemical synergistic effect through the “more light‐aiding, less bonding (LALB)” mechanism, providing a theoretical basis for the targeted design of anti‐cancer drugs. This technical platform not only establishes a quantitative evaluation system for the optimization of personalized photochemotherapy regimens but also expands the application of impedance sensing in drug development and precision medicine.

## 2 Results and Discussion

### 2.1 Experimental Platform

**Figure 1:**
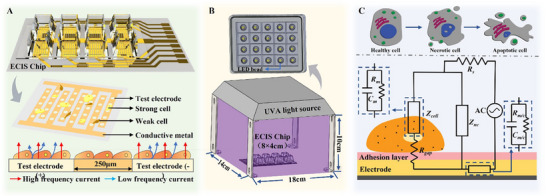
Evaluation system of cell survival state under UVA stimulation based on ECIS. A) Cell state and current effect on ECIS chip; B) System structure and lighting; C) Schematic diagram of ECIS impedance measurement under cell adhesion conditions.

We designed a combined chemo‐phototherapy system to explore and visualize the effects of cancer therapy using impedance spectrum, the principle and structure of which are shown in **Figure** **1**. The experimental platform is divided into 5 parts, namely impedance analyzer, data analysis, UVA irradiation, temperature control and cell culture. The electric cell‐substrate impedance sensing (ECIS) chip used in this study is an 8‐well array 8W10E+PET chip (Nanjing Applied Biophysics, China) with 8 square holes for cell culture. Each hole has 40 electrodes with a total electrode area of 1.96 mm^2^. An impedance analyzer (MFIA Impedance Analyzer, Zurich Instruments, Switzerland) is connected to the electrodes of the ECIS chip for impedance measurement, and real‐time impedance recording is performed using LabOne software (Zurich Instruments, Switzerland). Prior to impedance measurement, cells were stimulated using a homemade uniform UVA light source (LED Cold light source, Canyuan Optoelectronic Materials Co., LTD., China) with a wavelength of 395 nm and a power of 20 W. The light intensity was checked by a spectrometer (HR2000+ES, Ocean Insight, Shanghai) to ensure that the same light intensity was used for each experiment. The UVA lighting device covers the entire ECIS chip, and the battery is attached to the chip as Figure 1A. When tiny electrical signals are applied to the system at a relatively low frequency, most currents choose easy‐to‐pass bypass‐cellular and intercellular pathways. At high frequencies, it is sufficient to cause a rapid movement of electrons within the cell, moving back and forth between the membrane so that more current is selected to pass through the membrane (Figure 1A). The lighting device is a black box with a length of 18 cm, a width of 14 cm and a height of 10 cm. 20 LED lights with a power of 2 W are arranged on the plane as the light source. The lighting area is 252 cm^2^, and the total power is 20 W. An ECIS chip of ≈32 cm^2^ with 8 cell culture holes (8 cm^2^) is placed in the center of the device bottom plate. A heating platform is placed under the ECIS chip, which controls the temperature during the measurement and provides different temperatures for certain experiments (Figure 1B).

### 2.2 Impedance Measurement Pretreatment

**Figure 2:**
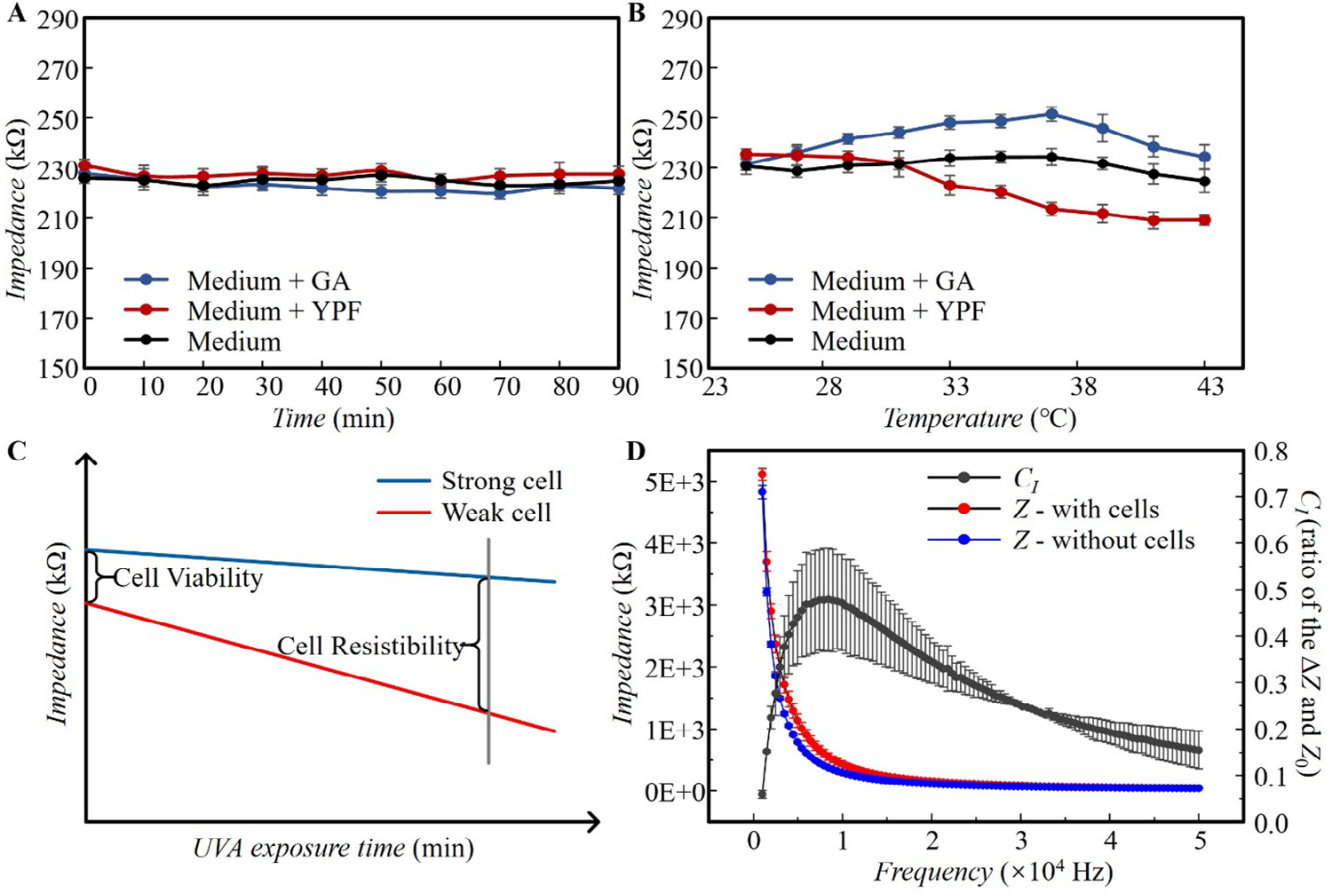
Impedance measurement preprocessing results. A) Impedance variation with time in different media; B) Impedance variation with temperature in different media; C) Schematic diagram of cell viability and resistibility assessment; D) The variation of impedance between with and without cells with frequency.

The Huh‐7 human liver cancer cells were cultured in DMEM complete medium supplemented with 10% fetal bovine serum and 1% penicillin/streptomycin solution in a flask placed in an incubator at 37 °C and 5% CO_2_. The medium was replaced every 2–3 days. Cell passage was carried out when the cells filled 80% of the flask. Then, 1.5 mL 0.25% trypsin was added to digest the adherent cells. When the cell edges were clear, new medium was added to terminate the digestion. The collected medium, including the cells, was centrifuged at 1000 rpm for 5 min, and the clear liquid at the top was removed. The cells were resuspended in new medium and counted with a blood cell counting plate, and then diluted to ≈2.5 × 10^5^ cells mL^−1^. A cell suspension of 300 µL was added to each well on the ECIS chip and cultured in an incubator for 24 h. After confirming that the cells were attached to the electrodes, the original medium was removed, and then the 300 µL new medium containing H_2_O_2_ or GA was added to the well. After being placed in the incubator for 3 h with H_2_O_2_ or 24 h with GA, the clear liquid in each well was removed by pipette sucking. Then, 300 µL of new medium was added for another 10 min of incubation before impedance measurement was performed. For impedance measurement, the electrode of the ECIS chip was connected to the impedance analyzer to record the real‐time impedance, transferred to a computer via USB for data analysis. The subsequent experiments were calibrated by the impedance value changing trend with time and temperature, and the results were shown in **Figure** **2**A,B. Live and dead cells have different adhesion ability as well as different cell membrane compounds and therefore different electric impedance. The direct impedance without UVA stress reflects cell viability, while the impedance change rate to the UVA doses indicates cell resistance, which is shown in Figure 2C.

To find the most sensitive frequency for impedance detection, a frequency sweep for the solution with and without cells was conducted from 0 to 50 kHz with a voltage of 30 mV. The impedance without cells gave the intrinsic impedance of the system and can be regarded as a background. As shown in Figure 2D, when increasing the signal frequency, the cell impedance decreased whether the cells were presented or not. This can be attributed to the cell polarization when the electric field at a lower frequency is applied. At low frequency, the cell membrane capacitance is in an open state with a large impedance, preventing the current from flowing in the extracellular region. When a high‐frequency electric field is applied to cells, the cell membrane is short‐circuited with a low impedance. Electric current easily flows through cells, causing a decreased impedance. Figure 2D shows that at any frequency in the sweeping range, the impedance of the solution with cells was significantly higher than that without cells. This may come from the cell membrane capacitance and the adhesion protein on the cells to the electrodes when the cells were presented. The difference between the impedance with and without cells was regarded as the cell impedance spectrum Δ*Z*, where Δ*Z = | Z – Z*_0_ *|*. Then the resolution of the cell impedance at a certain frequency can be expressed as *C*_I_ *=* Δ*Z / |Z*_0_ *|*, where *Z*_0_ is the impedance without cell adhesion. When *C_I_* reached the maximum value, the frequency *f_0_* was considered the most sensitive detection frequency. The sensitive frequency was affected by many factors, such as cell metabolism, culture medium consumption, intrinsic system electronic response, and so on. As can be seen from Figure 2D, *C_I_* reached a maximum at ≈10 kHz in our detection system. In all the subsequent experiments, 10 kHz was used as the detection frequency for the impedance measurement.

### 2.3 Effect of UVA Light Stimulation on Cellular Impedance

**Figure 3:**
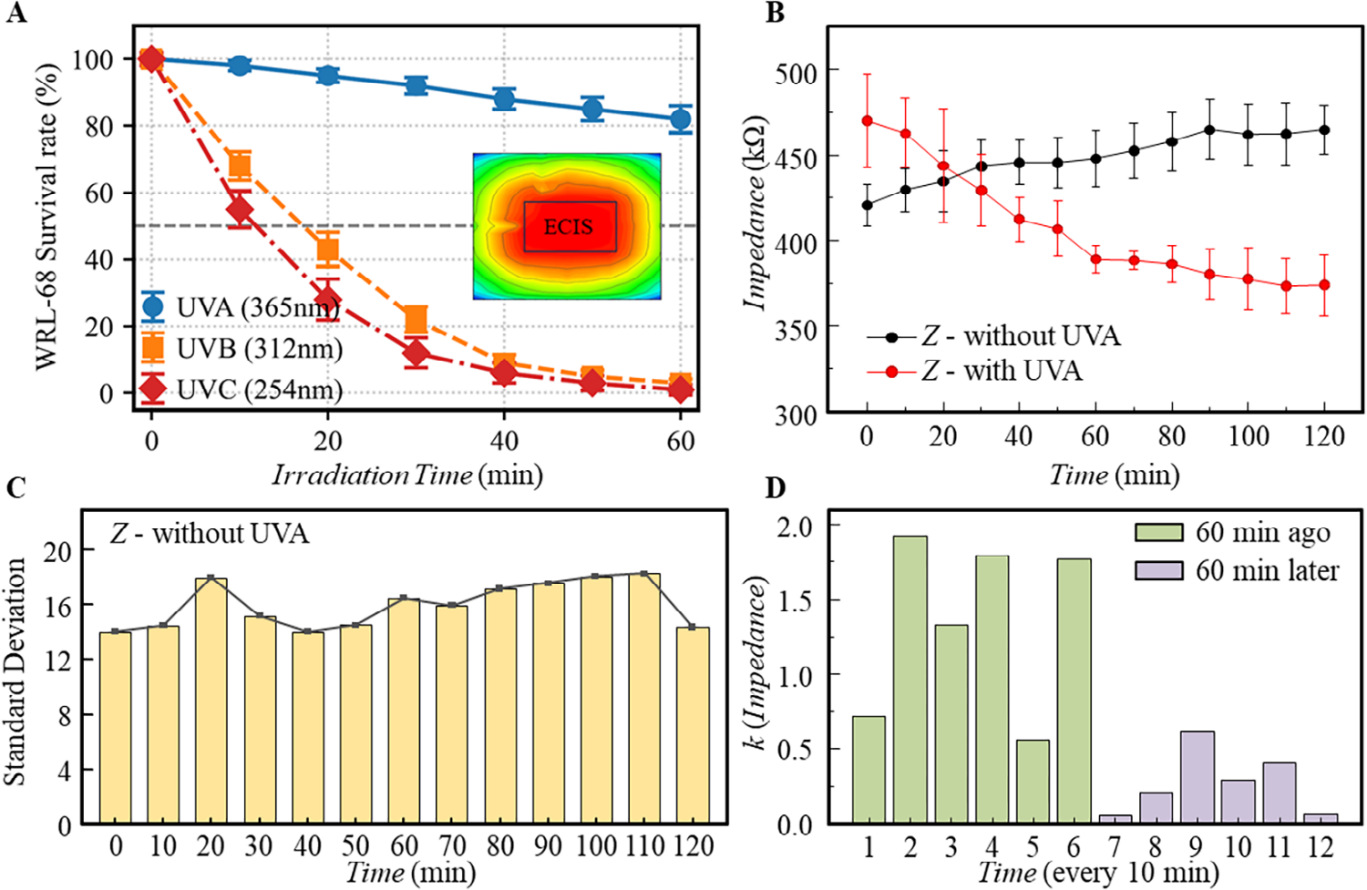
The range of UVA light stimulation and its effect on cellular impedance. A) The killing power of other ultraviolet bands on normal cells and plane projection of the light intensity distribution; B) Time‐dependent trends of cell impedance under UVA stimulation and shading treatments; C) Impedance standard deviation with time without UVA irradiation; D) Impedance change rate per 10 min under UVA irradiation.

To ensure uniform illumination among different holes on the ECIS chip, the light intensity was measured using a spectrometer at different positions 10 cm away from the light source on the base plate. The illumination intensity in the central area is uniform at ≈11 000 CD and gradually decreases to 4000 CD after reaching the edge of the device. There is a uniform area of ≈48 cm^2^ in the center, which is larger than the size of 32 cm^2^ of the ECIS chip. When the entire chip was placed at the center of the lighting device, the entire chip was exposed to the same level of light, providing equivalent stimulation to all 8 cell culture Wells on the chip. In order to determine the appropriate dose of light irradiation of appropriate intensity for stress cells rather than killer cells, the effects of ultraviolet light of different wavelengths under different irradiation times on cells were studied. As shown in **Figure** **3**A, under the condition of no drug intervention, UVB (312 nm) and UVC (254 nm) exhibited extremely strong killing effects on normal hepatocytes of WRL‐68: When exposed to UVB for 20 min, the cell survival rate dropped to 43 ± 5.1% (98.1 ± 2.7% in the nonexposed group), and further decreased to 22 ± 3.8% after 30 min. The toxicity of UVC was more intense, triggering cell apoptosis within 10 min, resulting in a sudden drop in the survival rate to 55 ± 5.5%, and only 28 ± 6.2% remained after 20 min. In contrast, under continuous irradiation for 60 min with UVA (365 nm), the survival rate of normal cells still remained at 82 ± 4.0%. Finally, UVA was determined for subsequent experimental exploration. The cells were divided into the light exposure group and the light exposure group. The sunshade group uses black tape to prevent UVA exposure. Two groups of cells were placed in the same ECIS chip under the UVA light source, and the impedance was recorded every 10 min for 2 h.

According to the black line in Figure 3B, the impedance of the cells without UVA exposure showed a slight upward trend that was caused by the cell proliferation, migration, and other behaviors of cells increased the number of adherent cells on the electrode. With reference to Figure 3C, the difference in standard deviation of impedance values over time is not significant under the blackout condition. It was proved that the impedance value was less affected by the cell growth condition under normal cell growth and the impedance value was less affected by cell growth. According to the red line in Figure 3B, the impedance of cells under UVA exposure decreased with elongated exposure time, and the decreasing trend gradually slowed down after 60 min. This decrease can be attributed to the cell stress from the UVA light. Under stress, a cell can be shocked to activate a heat shock response that generates proteins for protection, and therefore, alter the components in cytoplasm and cell membranes, causing the impedance drop. When the exposure was beyond the protection limit, cells would start the apoptosis progress, shedding the damaged cells from the electrode. With long enough irradiation time, most of the cells have shed from the electrode, slowing down the subsequent impedance decline. In Figure 3D, the impedance change rate before 60 min is significantly greater than after it. UVA irradiation time was less than 60 min, the cells were stimulated, but did not enter the apoptosis process. Therefore, in the subsequent experiments, we all chose 60 min as the light stimulation time.

### 2.4 Cell Viability and Resistibility Assessment Under UVA Stimulation

**Figure 4:**
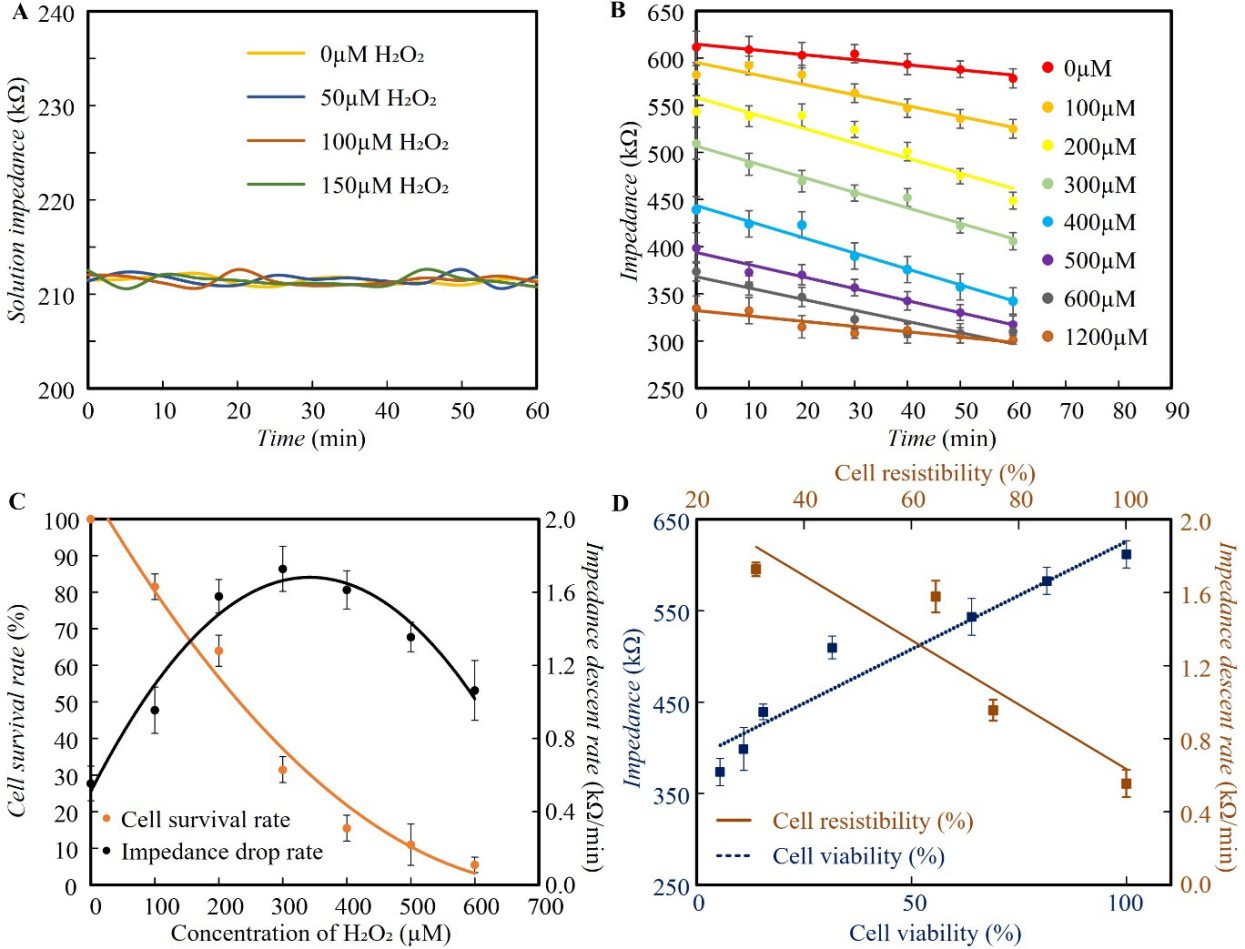
Evaluation of cell viability and resistibility by the UVA‐ECIS method. A) Impedance changes of H_2_O_2_ solutions with different concentrations (no cells) under UVA light stimulation; B) Impedance changes of cells treated with different concentrations of H_2_O_2_ under the UVA light stimulation; C) The relationship between cell impedance descent rate and cell survival under the UVA light stimulation; D) Calibration of the relationship between impedance and cell viability compare with traditional methods, Calibration of the relationship between impedance descent rate and cell resistibility compare with traditional methods.

To validate the performance of our ECIS system under UVA stimulation for simultaneous assessment of cell viability and resistibility, a series of experiments was conducted using Huh‐7 liver cancer cells treated with varying concentrations of hydrogen peroxide (H_2_O_2_), a compound known to affect cell vitality. To eliminate the potential influence of UVA illumination on the impedance of the medium itself, we measured the impedance of H_2_O_2_‐containing media in the absence of cells under different levels of UVA exposure. As shown in **Figure** **4**A, the impedance of the cell‐free medium remained stable across all H_2_O_2_ concentrations and UVA intensities. These results confirm that any observed impedance changes in subsequent experiments originate from cellular responses rather than alterations in the medium.

As shown in Figure 4B, the initial cell impedance without UVA exposure decreased with increasing H_2_O_2_ concentration. The static impedance was ≈600 kΩ in the absence of H_2_O_2_, but it dropped to 300 kΩ when 1200 µm H_2_O_2_ was applied. This indicates that static impedance effectively reflects cell viability under oxidative stress conditions such as H_2_O_2_ treatment. Upon UVA exposure, the cell impedance further decreased due to UV‐induced cell damage. As illustrated in Figure 4C, the rate of impedance decline was dependent on the H_2_O_2_ concentration. When the concentration was below 300 µm, the impedance decline rate under UVA exposure was positively correlated with the H_2_O_2_ level. However, further increases in H_2_O_2_ concentration led to a reduction in the impedance descent rate. Specifically, for cells treated with 1200 µm H_2_O_2_, the impedance remained unchanged during UVA exposure. This is attributed to the extremely low number of viable cells; when the number of live cells on the electrode surface becomes negligible relative to dead cells, their contribution to the total impedance is overshadowed. As shown in Figure 4C, the cell survival rate dropped to below 20% when the H_2_O_2_ concentration exceeded 300 µm. At 1200 µm, nearly all cells were dead, resulting in no further impedance change upon UVA illumination. Based on this mechanism, static impedance and the rate of impedance decline under UVA exposure were used as calibration parameters to quantify cell viability and resistibility, respectively. The ECIS‐UVA calibration curves for measuring cell viability and resistibility are presented in Figure 4D. A positive linear correlation was observed between cell viability and static impedance, while cell resistibility also exhibited a linear relationship with the impedance decline rate under UVA irradiation.

### 2.5 Reliability Verification and Comparative Analysis

To validate the feasibility of our definition, we compared our methods with the traditional gold standard methods, trypan blue staining for cell viability and MTT assay and cell resistibility assessment. For Trypan blue staining, the cultured cells were digested with 1.5 mL 0.25% trypsin for 3 min to prepare the cell suspension. The supernatant was discarded after centrifugation at 1000 rpm for 5 min. Then, 1 mL of the new culture medium was added to suspend the cells. Subsequently, 100 µL of trypan blue dye was added for staining. Within 1 min, 10 µL of the stained cells were transferred to a cell counting plate for observation under a microscope (xsp‐63x) (Shanghai Optical Instrument Factory, China). The number of unstained living cells and stained dead cells was counted within 3 min. Defined here: Cell viability = Number of living cells/total number of cells×100%. For the MTT method, the cells were inoculated onto 48‐well culture plates with 300 µL per well. After being cultured in the incubator until the cells adhered to the wall, the original medium was removed, and then 300 µL of new medium containing GA or YPF was added to the culture for 24 h. Then, 20 µL MTT solution (5 g L^−1^) was added into each well. Having been continually cultured in a CO_2_ incubator for 4 h, the upper solution was carefully sucked away. DMSO of 150 µL was added and kept in each well for 10 min. The absorbance of each well was thereafter recorded using an enzyme‐linked immune monitor at an excitation wavelength of 570 nm. Defined here: Cell resistibility = OD value in the dosing group/OD value in the control group×100%.

**Figure 5:**
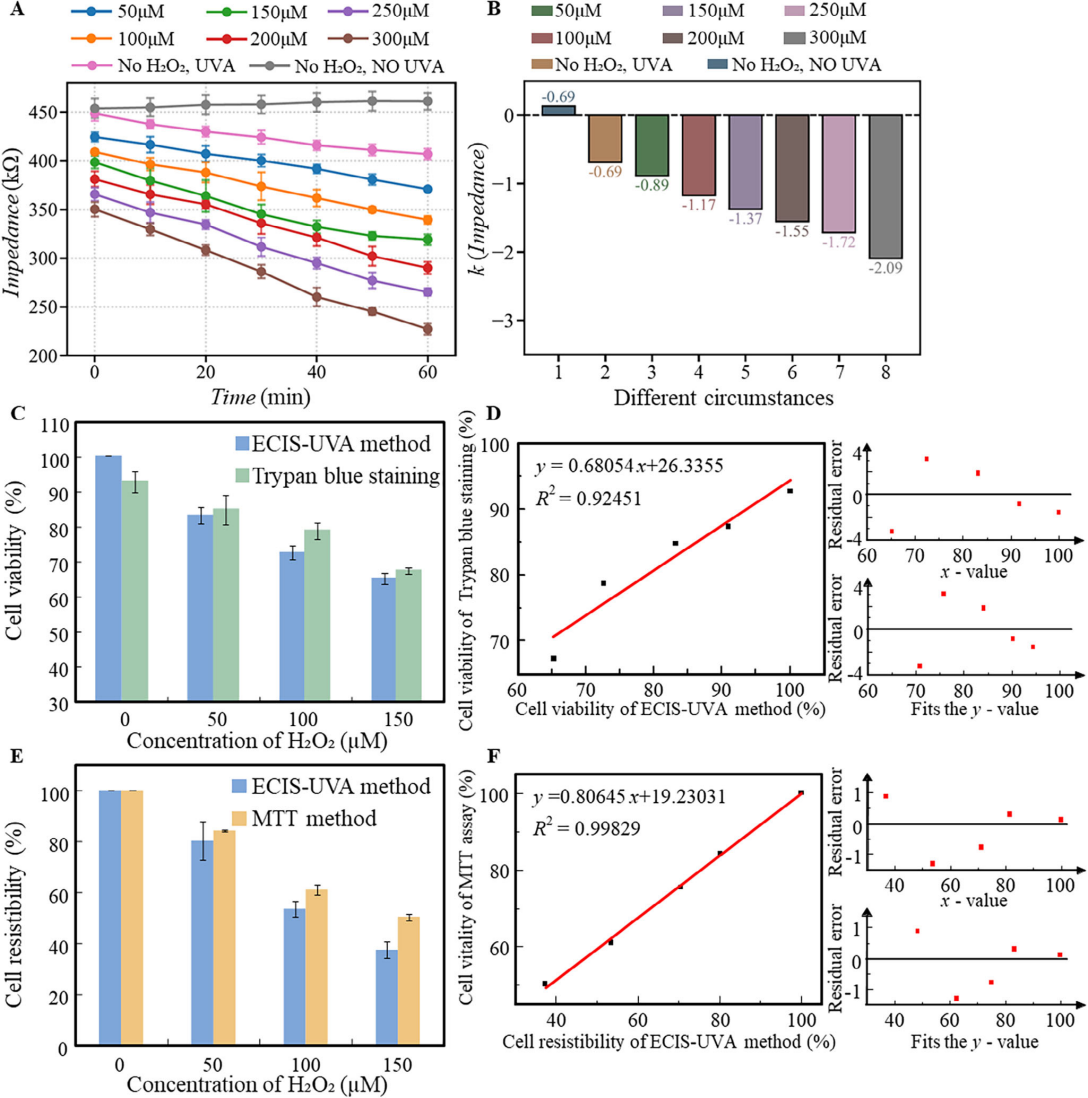
Cell activity measured by UVA stimulation impedance sensing after adjustment with hydrogen peroxide. A) Changes in impedance of cells cultured with different concentrations of hydrogen peroxide under UVA stimulation; B) Change of impedance slope under different conditions; C) Comparison of the cell viability measured by the impedance value at 0 point of optical stimulation in Figure (A) and the cell viability measured by the trypan blue method; D) Fitting result of ECIS‐UVA method and Trypan blue staining method; E) Comparison of the cell resistibility as measured by impedance descent rate in Figure (A) and the cell resistibility as measured by MTT; F) Fitting result of ECIS‐UVA method and MTT method.

**Figure** **5**A shows the impedance responses of cells treated with H_2_O_2_ at concentrations ranging from 0 to 300 µm under UVA illumination. Parallel control samples, prepared under identical chemical conditions but shielded from UVA exposure, were tested on the same ECIS system to assess impedance changes in the absence of illumination. Figure 5B illustrates the impedance slope variations under different conditions. A more severe treatment condition results in a more rapid decline in impedance values. Figure 5C,E compares the cell viability and resistibility, respectively, as measured by the ECIS‐UVA method and conventional methods. Upon H_2_O_2_ treatment, the linear correlation coefficient between the ECIS‐UVA method and the Trypan Blue staining method was 0.9375, and that with the MTT assay was 0.8947. Figure 5D shows the linear regression between the ECIS‐UVA and Trypan Blue methods, yielding an *R* ^2^ value of 0.92451. Figure 5F displays the linear regression between the ECIS‐UVA and MTT methods, with an *R* ^2^ value of 0.99829.

### 2.6 Method Verification

Given the convenience of our approach for cell viability and resistibility assessment from a single ECIS measurement under UVA exposure, it can be used to monitor the cell responses to drugs in cancer drug screening. Here, we used commercially available liver cancer cells (Huh‐7) as the model cancer cells, and GA as the model drugs, liver normal cells (Wrl‐68), and drug YPF as counterpart controls to investigate the drug screening on the ECIS chip. GA was reported to have anticancer effects by immunomodulation and inhibiting liver inflammation. YPF was mainly used for the treatment of respiratory disease, and its effect on cancer cells is mild, so it was used as a control with GA. Each drug was dissolved in DMSO and then diluted in the DMEM medium to the final concentrations. Liver cancer cells were inoculated on the electrode on the 8W10E+PET ECIS chip at the ≈1.25 × 10^5^ cells cm^−2^. After the cells were completely adhered, GA or YPF of different concentrations was added and cultured for 24 h. Before the impedance measurement, the supernatant was sucked away, and 300 µL of new medium was added. The cells were incubated at 37 °C and 5% CO_2_ for 10 min. Then, the UVA stimulation was applied, and the impedance was measured. Here, our main purpose is to verify whether the method can distinguish the toxicity of different drugs on cells; the mechanism of drug action on cells is not explored.

**Figure 6:**
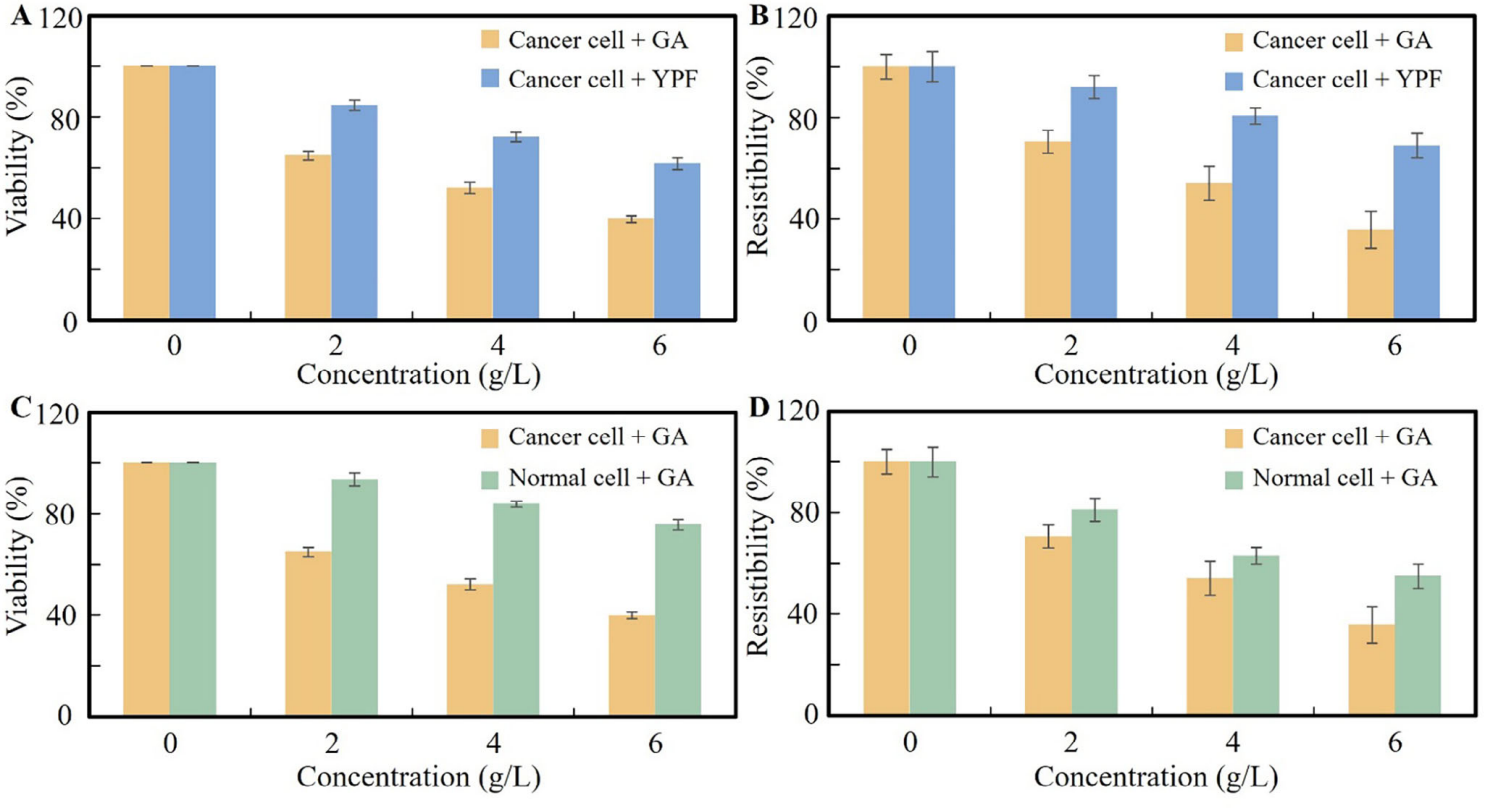
Comparison of cell viability and resistibility after the addition of different drugs. A) Viability: The cancer drug GA and the common drug YPF act on the same cancer cells for 24 h; B) Resistibility: The cancer drug GA and the common drug YPF act on the same cancer cells for 24 h; C) Viability: The anticancer drug GA acts on liver cancer cells and normal liver cells for 24 h; D) Resistibility: The anticancer drug GA acts on liver cancer cells and normal liver cells for 24 h.

**Figure** **6**A,B illustrates the effects of GA and YPF on cancer cell viability and resistibility, respectively. As shown, treatment with GA resulted in a marked reduction in both viability and resistibility of cancer cells as the drug concentration increased. Specifically, at a concentration of 6 g L^−1^, cell resistibility decreased from 100% (untreated) to 35%, while cell viability declined to 40%—below the 50% threshold typically used to indicate effective anticancer activity. These results suggest that GA possesses cytotoxic effects on cancer cells, in addition to its known anti‐inflammatory properties in normal cells. In contrast, treatment with YPF led to only a slight reduction in cell viability, with minimal impact on resistibility. Both parameters remained above 60% compared to untreated controls. This outcome is consistent with YPF's traditional use as a mild immunomodulatory agent for cold prevention, indicating that its cytotoxicity is negligible. Unlike GA, YPF does not exhibit significant pro‐apoptotic effects on cancer cells.

Figure 6C,D compares the viability and resistibility of both cancer and normal cells following GA treatment. Cancer cells showed a pronounced response to GA, while normal cells exhibited relatively stable viability and resistibility. In particular, the viability of normal cells remained above 75% even at 6 g L^−1^ GA. However, resistibility in normal cells decreased to 55% at this concentration, although this reduction was less severe than that observed in cancer cells, where resistibility dropped to 30%. These findings indicate that GA exerts cytotoxic effects on both cancer and normal cells, but with greater selectivity and potency toward cancer cells—particularly liver cancer cells. Nonetheless, the data also suggest some level of toxicity to normal cells at higher GA concentrations. Overall, these experimental results demonstrate that the ECIS‐UVA method can effectively differentiate the cytotoxic effects of various compounds on cell populations, highlighting its potential as a reliable tool for drug screening.

To comprehensively evaluate the drug concentration effect, we expanded the detection concentration range of GA and YPF to the 0–10 g L^−1^ interval outside the clinically recommended dose and set up three independent experimental replicates at each concentration point. As shown in Figure S2 (Supporting Information), the experimental results revealed significant differences in dose selectivity: At a concentration of 10 g L^−1^, the inhibition rate of GA on Huh‐7 hepatoma cells reached 71.6%, and the survival rate of cancer cells was 28.4 ± 3.2%, which was significantly lower than that of normal hepatocyte WRL‐68 at 73.8 ± 4.3%, and the statistical difference reached the level of *p* < 0.001. The dose response slope of cancer cells was −7.2 ± 0.5% gL^−1^, which was 3.4 times that of normal cells −2.1 ± 0.3% gL^−1^, indicating that GA has stronger targeting selectivity. At the same concentration, YPF reduced the survival rate of Huh‐7 to 49.8 ± 3.5%, which was 34.2% lower than that of normal cells (75.2 ± 4.6%). The statistical difference was *p* < 0.01. Its dose response slope was −5.2 ± 0.4% gL^−1^, which was significantly lower than the cytotoxic efficiency of GA, suggesting that there was an essential difference in the mechanism of action between the two. Although the high‐concentration effect has limited applicability in current clinical scenarios, these findings provide new ideas for the modification of anti‐tumor drugs.

### 2.7 Drug Screening Assessed with ECIS Measurements

In the above section, simple drugs were used to confirm the applicability of ECIS‐UVA method for drug screening, and then further verification was carried out for the combination of Huh‐7 and Wrl‐68 anti‐liver cancer drugs. Anti‐cancer drugs are required to be less lethal to normal cells and more lethal to target cancer cells. The impedance numerical evaluation criteria are consistent with the previous ones; that is, the more normal adherent cells, the larger the impedance value, and the more suspended dead cells, the smaller the impedance value. We numbered the eight holes of ECIS chip in Figure 1A from top to bottom and from left to right as 1–8, respectively (the drug concentration and status of each hole were described by numbering later). The concentration of cell solution was ≈1.54 × 10^6^ PCS mL^−1^, and 350 µL was added to each well and cultured for 24 h until cell adhesion. We tested four drugs that are currently targeted for liver cancer, Lenvatinib (E7080), Cabozantinib (BMS‐907351), Sorafenib (BAY 43–9006), and Regorafenib (BAY 73–4506), purchased from Aladdin. The four drugs mentioned above are insoluble in water. The drug with an initial concentration of 10 mm was dissolved in dimethyl sulfoxide (DMSO) and subsequently diluted in medium. Because DMSO has certain toxicity to cells, two concentrations of DMSO (Xinyu Biotech Co., Ltd.) solutions (10^−4^ and 10^−5 ^mol) were selected as controls in the pre‐experiment. The proportion of anticancer drugs added to the cell solution was ≈ 10 µL per 10 000 cells, and the drug was assigned with 6 concentration gradients, namely 1, 2, 4, 8, 10, and 100 µm, of which 100 µm was the lethal dose. Drug screening experiments were carried out in the culture chamber of the ECIS chip, with ≈5 × 10^3^ cells inoculated per well. The chips were transferred to a CO_2_ incubator at 37 °C for 24 h, after which the drug was inoculated.

**Figure 7:**
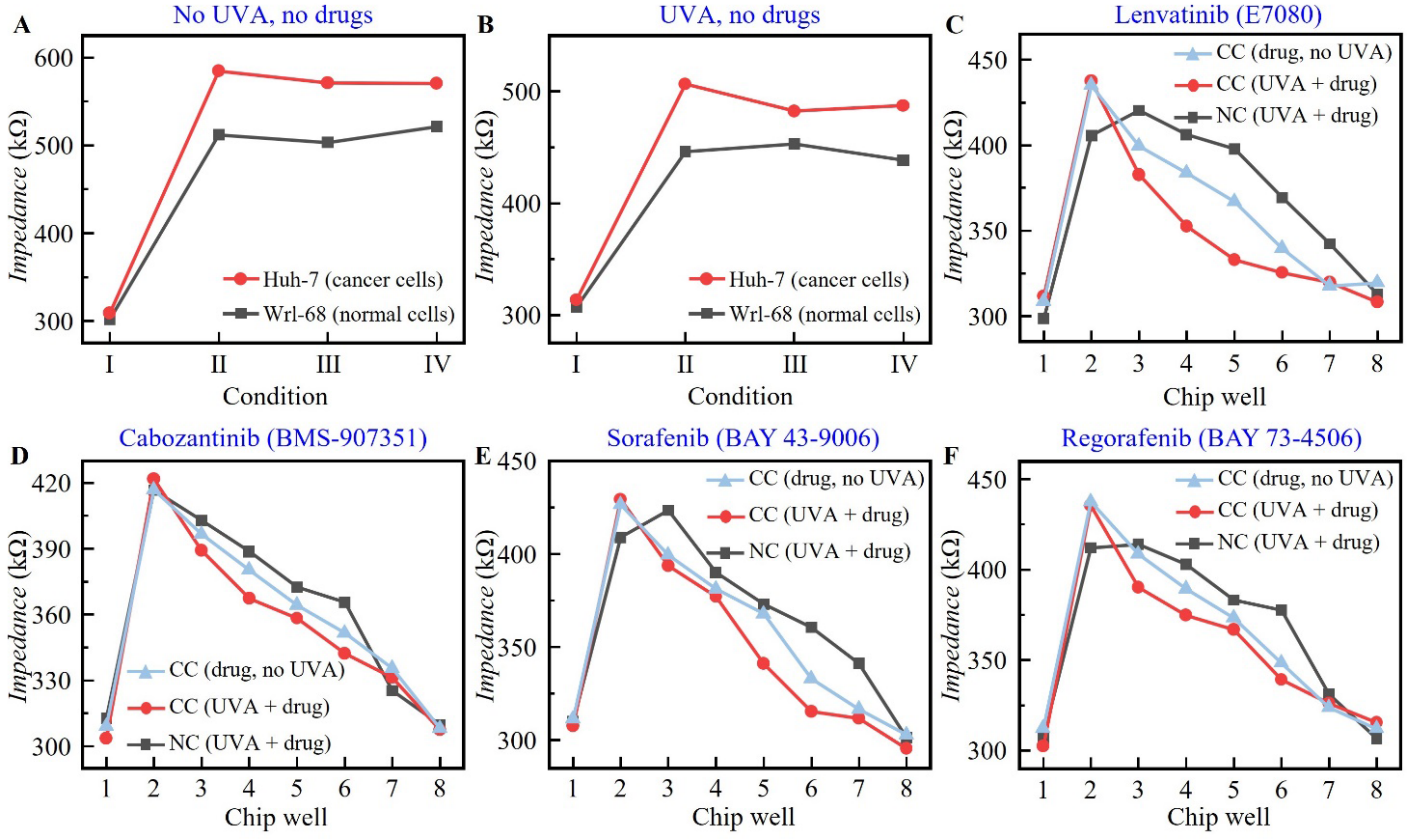
Drug screening results of ECIS‐UVA method (1 well of ECIS chip was the medium as the control, 2–8 wells were cells cultured for 24 h to the wall, 2 well did not add drugs, 3 well added 1 µm drug, 4 well added 2 µm drug, 5 well added 4 µm drug, 6 well added 8 µm drug, 7 well added 10 µm drug, 8 well added 100 µm drug). A) No UVA, no drugs‐ control group 1; B) UVA no drugs‐ control group 2; C) Screening results of Lenvatinib at different concentrations; D) Screening results of different concentrations of Cabozantinib; E) Screening results of Sorafenib at different concentrations; F) Screening results of different concentrations of Regorafenib; (In Figure C–F, CC is cancer cell, NC is normal cell).

**Figure 8:**
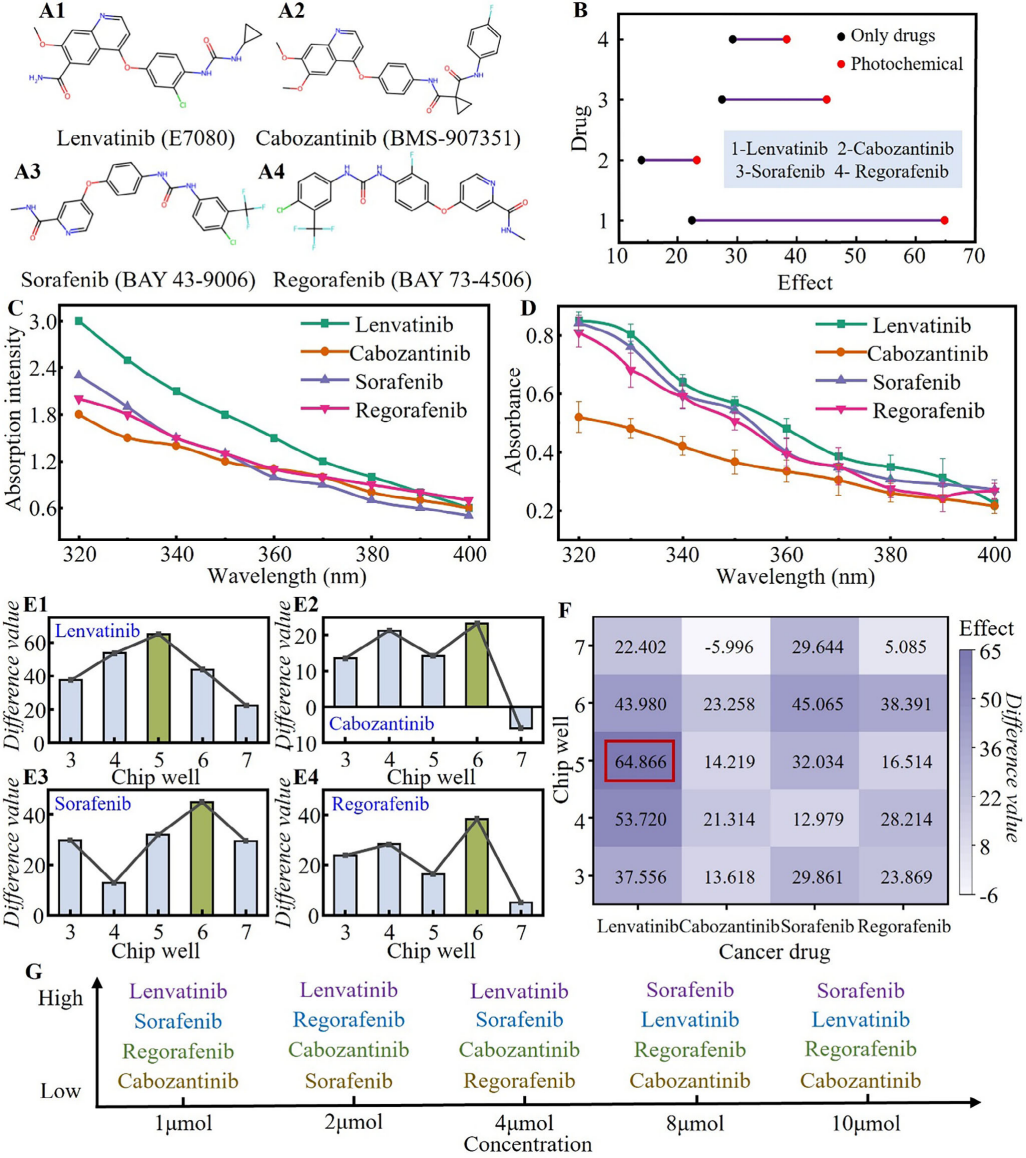
Analysis of drug sieve results based on the principle of photochemical interaction. A) Chemical formula for four drugs; B) Comparison of anticancer effects of only drug and photochemical coupling; C) Estimated absorption intensity of four drugs in UVA band; D) Measurement of absorbance of four drugs in UVA band; E) Drug screening effect in the normal dose range of 3–7 wells (the difference between the resistance of normal cells and cancer cells under UVA); F) Combined results of four drug screens; G) The screen effect of different concentrations of drugs is displayed, in which purple has the best effect, blue is the second, green is the third second, and yellow is the worst.

To confirm the outstanding contribution of the interaction of phototherapy and chemotherapy in cancer treatment, excluding the effect of UVA on cells alone, we set up control groups without and with UVA irradiation in the absence of anticancer drugs (control group 1 and control group 2, respectively). The 1–4 wells of the chip are group Wrl‐68, and the 5–8 wells are group Huh‐7. Among them, 1 and 5 correspond to no cells in the medium (corresponding to cases I in **Figure** **7**A,B), 2 and 6 correspond to cells cultured under normal conditions (corresponding to cases II in Figure 7A,B), and 3 and 7 add 10 µL and 10–4 mol of DMSO (corresponding to cases III in Figure 7A,B), 4 and 8 in Figure 7A and B were added with 10 µL and 10–5 mol of DMSO (corresponding to cases IV in Figure 7A,B). Figure 7A,B shows the experimental results of control groups 1 and 2. Compared with normal cells, cancer cells have a faster growth rate, more adherents and larger impedance. It can be seen from the experimental data added with DMSO that a small amount of DMSO has almost no influence on the experimental process. According to the experimental data of the two control groups, only UVA irradiation has a certain killing effect on cells, but it is not the leading effect. The killing effect on normal cells and cancer cells is almost the same, and no obvious anticancer effect is seen.

Figure 7C–F shows the impedance results of Lenvatinib, Cabozantinib, Sorafenib and Regorafenib combined with UVA for 60 min, respectively. In the ECIS chip, 1 well was the medium as the control, 2–8 wells was the cells cultured for 24 h to the wall, 2 well was without drug, 3 well was added with 1 µm drug, 4 well was added with 2 µm drug, 5 well was added with 4 µm drug, 6 well was added with 8 µm drug, 7 well was added with 10 µm drug, 8 well was added with 100 µm drug. Since anticancer drugs need to make normal cells survive a large amount and cancer cells have a high mortality rate, it can be seen that the four common anticancer drugs on the market all meet the above conditions, indicating that our proposed method has a certain reliability. Figure 7C–F compares the results of the trial using only drugs, and it can be seen that the results of phototherapy‐driven chemotherapy are due to the results of anti‐cancer drugs only.

### 2.8 Analysis of Drug Screening Results

**Figure** **8**A shows the chemical formulas of four anticancer drugs. Lenvatinib has the least number of conjugated chemical bonds, while Cabozantinib has the most conjugated chemical bonds. Figure 8B shows the killing effect of four drugs on cancer cells. Black is the effect value under the action of drugs only, and red is the effect value under the action of photochemical coupling. The anti‐cancer effects of Lenvatinib, Cabozantinib, Sorafenib and Regorafenib were 22.42, 13.96, 27.48, and 29.28, respectively. The anti‐cancer effects of phototherapy‐driven chemotherapy were 64.87, 23.25, 45.07, and 38.39, respectively. The greater the difference, the more obvious the photoassisted effect of the drug. The energy transition values of Lenvatinib, Cabozantinib, Sorafenib and Regorafenib are ≈4.6, 3.85, 4.25, and 4.27, respectively. Therefore, the photochemical interaction of Lenvatinib before the excited state is the most durable.

To preliminarily validate the accuracy of our conclusions, we evaluated the UVA absorption properties of the four compounds (Lenvatinib, Cabozantinib, Sorafenib, and Regorafenib) through chemical morphology‐based simulations and experimental absorbance measurements. Molecular orbital simulations within the 320–400 nm wavelength range revealed distinct absorption trends correlated with structural features. Lenvatinib exhibited significantly higher absorption intensity than Cabozantinib across most wavelengths (Figure 8C). This disparity aligns with their molecular configurations: Cabozantinib's extended conjugated system enhances π‐electron delocalization, redshifting its maximum absorption peak and reducing absorption intensity within the 320–400 nm range. In contrast, Lenvatinib's smaller conjugated system positions its absorption peak closer to this UV band, resulting in stronger absorption. Additionally, hydroxyl groups in Lenvatinib facilitate electron transfer, further amplifying its light absorption capacity, whereas Cabozantinib lacks analogous electron‐donating moieties. While these simulations provide qualitative trends rather than quantitative accuracy, they corroborate our earlier findings.

Spectrophotometric absorbance measurements further clarified the compounds' photochemical potential: Lenvatinib demonstrated superior broadband absorption (0.849 at 320 nm → 0.224 at 400 nm), with a steady decline rate indicating sustained photochemical activity across the UVA spectrum. This broad‐spectrum absorption suggests optimal utility in photo‐assisted chemotherapy. Cabozantinib showed minimal absorption (0.519 at 320 nm → 0.215 at 400 nm) with a shallow decline, reflecting negligible photo‐enhancement effects. Sorafenib displayed intermediate absorption (0.84 at 320 nm → 0.272 at 400 nm), characterized by a gradual decline, implying moderate but stable photochemical contributions. Regorafenib exhibited wavelength‐dependent absorption, with a sharp decline from 0.809 at 320 to 0.266 at 400 nm, indicating strong short‐wavelength activity (320–350 nm) but limited utility at longer wavelengths. Lenvatinib emerges as the most effective candidate for photo‐assisted chemotherapy due to its robust broadband absorption and gradual intensity decay. Cabozantinib's weak absorption underscores its limited photochemical utility, while Sorafenib and Regorafenib demonstrate intermediate profiles—the former offering stable absorption and the latter excelling in short‐wavelength UV regions. These experimental and computational results collectively reinforce our initial conclusions regarding structure‐dependent photochemical behavior.

If expressed by impedance value, Figure 8E shows the impedance difference between normal cells and cancer cells under UVA action of anti‐cancer drugs in the normal dose range of Wells 3–7. The greater the difference, it indicates that the combination of the drug and phototherapy has the best effect. The optimal dose of each anti‐cancer drug under UVA action is intuitively shown in the histogram. The best concentration of Lenvatinib was 4 µm, and the best concentration of Cabozantinib, Sorafenib and Regorafenib was 8 µm. Figure 8F shows the comprehensive results of the four drugs. It can be seen that 4 µm Lenvatinib combined with phototherapy has the best therapeutic effect, 8 µm Sorafenib and Regorafenib combined with phototherapy has the second therapeutic effect, and 8 µm Cabozantinib has a slightly weaker therapeutic effect. The killing effect of cancer cells was evaluated by the combined impedance value. The cancer treatment effect of light therapy alone was 5.14. Combined with Figure 8B, it can be seen that phototherapy has a certain driving effect on chemotherapy and can achieve the effect of one plus one is greater than two. The driving effect of Lenvatinib is the most obvious. The reason for this is that the more conjugated chemical bonds, the stronger the UV absorption peak is usually produced, and the stronger the UV absorption is. The UVA we use is relatively mild, and the irradiation time is short, that is, it is within the excitation threshold. The more conjugated chemical bonds within the excitation threshold, the more stable the molecule is usually, that is, the fewer conjugated chemical bonds, the more obvious the light‐aiding properties. The conjugated chemical bonds of Lenvatinib are the least; the photoadjuvant effect is obvious, and the drug combined with phototherapy has the best effect on cancer treatment. The results of the drug screening are visually shown in Figure 8G.

Through an in‐depth study, we propose the more light‐aiding, less bonding (LALB) mechanism, meaning less conjugated chemical bonding within the excitation threshold results in more pronounced light‐aiding properties. For phototherapy‐driven chemotherapy for cancer treatment, since more conjugated chemical bonds usually result in stronger UV absorption peaks, the more conjugated chemical bonds a molecule has, the stronger its UV absorption will be. This absorption may lead to the formation of excited states of the molecule and even trigger further photochemical reactions. However, the UVA we used is relatively mild, and the irradiation time is short, which is not enough to cause electronic transition, photolysis reaction, etc., and does not reach the excitation threshold. Within the excitation threshold is significantly different from the situation after the above excited state. The greater the number of conjugated chemical bonds within the excitation threshold usually makes the molecule more stable, that is, the fewer conjugated chemical bonds, the more obvious the photoaid. The analysis of the above principles fits well with the results shown in Figure 8A. Lenvatinib has the least number of conjugated chemical bonds and obvious photoassistance, and the drug combined with phototherapy should have the best effect on cancer treatment. Cabozantinib has the highest number of conjugated chemical bonds, and the photoaid effect is weaker, the therapeutic effect of this drug under the action of phototherapy should be lower than that of the other three drugs. Above all, the theoretical analysis results based on the principle of photochemical interaction are in good agreement with the experimental results.

## 3 Conclusion

We employed UVA stimulation combined with relevant drugs to simulate a prolonged chemotherapy‐phototherapy combination treatment. Two parameters—cell viability and resistibility—were quantified using impedance measurements with an ECIS chip to visualize tumor cell status during treatment. A model correlating impedance changes with cancer cell survival was established. After optimizing system parameters, the ECIS measurement frequency was set to 10 kHz, the UVA wavelength to 395 nm, and the light stimulation duration to 60 min. To verify model accuracy, we compared our results with two gold‐standard assays—Trypan blue staining and the MTT method—achieving linear correlations of 93.75% and 89.47%, respectively. Although absolute values of viability and resistibility showed slight differences from traditional methods, the overall trends were consistent. This discrepancy likely arises because each method targets different cellular aspects: Trypan blue assesses membrane permeability, MTT measures intracellular enzyme activity, and UVA stimulation reflects changes in cellular heat shock proteins. Despite these differences, relative cell resistance indices remained comparable across conditions. These findings validate ECIS‐UVA as a feasible approach for cell state assessment, demonstrated here in drug screening experiments using liver cancer cells and GA as a model drug, accurately distinguishing drug effects. Building on this, four anticancer drugs with notable efficacy were selected for further screening. We propose the “Light‐Aiding, Less Bonding” (LALB) mechanism, whereby fewer conjugated chemical bonds within the excitation threshold enhance phototherapy efficacy. Photochemical interaction experiments confirmed that phototherapy can potentiate chemotherapy effects.

In summary, by monitoring cancer cell impedance changes induced by UVA, we visualize drug screening processes to identify effective anticancer agents, tailor personalized treatments, and develop new drugs.^[^ ^63^, ^64^, ^65^ ^]^ Furthermore, exploring cancer cell responses to UVA stimulation provides a foundation for novel phototherapy methods or photosensitizers,^[^ ^66^ ^]^ which may offer improved targeting with reduced toxicity and represent a promising future direction in cancer treatment.^[^ ^67^, ^68^, ^69^ ^]^

## 4 Experimental Section

### Preparation of Experimental Materials

The Huh‐7 human liver cancer cells were from Xinyu Biotechnology Co., Ltd (Shanghai). The cell culture medium contained Dulbecco's modified eagle medium (DMEM), 10% fetal bovine serum (FBS), and 1% penicillin/streptomycin solution (P/S), which were from Gibco (USA). The 0.25% trypsin ethylenediamine tetra‐acetic acid (EDTA) came from Life Technologies GmbH (Germany). The H_2_O_2_ stock solution at 30% was purchased from LIRCON (Shandong, China) and then diluted to various concentrations in the cell culture medium in different experiments. The Glycyrrhizic Acid (GA) was from Shanghai Minrell Chemical Technology Co., Ltd., and the Yu‐Ping‐Feng (YPF) powder was from Jibel Pharmaceutical Co., Ltd (Jiangsu). GA and YPF were initially dissolved in dimethyl sulfoxide (DMSO) for stock solutions of 10 g L^−1^ and diluted to various concentrations in the cell culture medium for experiments. The MTT solution (5 g L^−1^) and the trypan blue solution (0.4%) were purchased from Voridas (Nanjing, Jiangsu). Screening drugs were Lenvatinib (E7080), Cabozantinib (BMS‐907351), Sorafenib (BAY 43–9006), Regorafenib (BAY 73–4506), purity 10 mm, purchased from Aladdin.

### Selection of the UVA Light

UV light as a stress factor has been investigated to show genetic or nongenetic cell damage. The toxic effect of UV light on cells mainly depends on the wavelength, where UVC (200–280 nm) and UVB (280–315 nm) have the strongest ability to induce cellular DNA damage in the absorption band of cellular DNA. Yet, the main reason for UVA (315–400 nm) induced cell damage is to induce cells to produce reactive oxygen species (ROS) and inhibit cell growth. This damage is nongenetic and easier to repair than direct DNA damage. Therefore, it could be used as a safe external stress factor. To test the cell tolerance to UVA stress, 395 nm UVA was chosen as the light source for its mild stimulation of the cells. On the one hand, it can simulate the damage to cancer cells, and on the other hand, reduce the damage to normal cells as much as possible.

### Optimization of the Electric Signal for Impedance Measurement

In the ECIS system, the impedance comes from the cell culture medium, adhered cells, matrix/coating, and electrode‐electrolyte interface. The equivalent electric circuit is shown in Figure 1C. In this system, cell membranes are often viewed as capacitors due to the structure of the lipid phosphate bilayer. The cytoplasm is regarded as a resistor reflecting the barrier function of the cell conductance. When cells attached to the exposed electrode proliferate or die, it alters the membrane and cytoplasm conditions and the flow of current changes, resulting in a change in the impedance of the whole system. Therefore, measuring changes in impedance can reflect the health status of the attached cells. To minimize the impairment of the electric signal on cells, the alternating current is normally used in ECIS measurement, which is defined by parameters of voltage and frequency.

In Figure 1C, when an AC signal is applied, electrical current flows both through cells via *Z*_c_ *(ω)* and around the cell via *Z*_nc_ *(ω)*. *Z*_nc_ *(ω)* refers to the interface equivalent impedance of the cell‐free region; *Z*_c_ represents the interface equivalent impedance of the cell‐covered area. They are related to the adhesion area A, which can be expressed as:

(1) $$Z_{c}\left(\omega \right)=\frac{A_{\mathrm{cell}}}{A}Z\left(\omega \right)+Z_{\mathrm{cell}}$$

(2) $$Z_{\mathrm{nc}}\left(\omega \right)=\frac{A-A_{\mathrm{cell}}}{A}Z\left(\omega \right)$$

where *Z*_cell_ is the cell impedance composed of membrane capacitance and membrane resistance. *Z(ω)* represents the equivalent impedance of the electrode‐electrolyte interface and region *A*, which can be expressed as:

(3) $$Z\left(\omega \right)=Z_{\mathrm{CPE}}+R_{\mathrm{ct}}$$

where *R*_ct_ is charge transfer resistance, usually regarded as pure resistance. The constant‐phase element CPE is used to characterize the nonlinearity of frequency‐dependent electrical double‐layer impedance in the electrode–electrolyte interface, and CPE can be expressed as

(4) $$Z_{\mathrm{CPE}}=1/\left[Q{\left(j\omega \right)}^{n}\right]$$

Angular frequency *ω = 2πf*, where *f* is the alternating current frequency provided by the impedance analyzer. n is a constant (0≤*n*≤1), *Q* is the magnitude of *Z*_CPE_, and *j* denotes the imaginary number unit given as $\sqrt{-1}$. A constant n close to 1 indicates stronger capacitance characteristics, and close to 0 indicates stronger resistive characteristics.

Therefore, under the action of AC electrical signals, the overall impedance of the system can be expressed as:

(5) $$Z_{\mathrm{total}}=Z_{\mathrm{nc}}\left(\omega \right)//\left[Z_{c}\left(\omega \right)+R_{\mathrm{gap}}\right]+R_{s}+Z_{m/c}$$

where *Z*_nc_ *(ω)* refers to the interface equivalent impedance of the region without cell coverage; *Z*_c_ *(ω)* represents the interfacial equivalent impedance of the cell‐covered area. *R*_solution_ is the resistance of culture medium; *Z*_m/c_ is matrix/coating impedance; *R*_gap_ is intercellular resistance.

The overall impedance of the system is affected by many factors. At a high detection frequency, the overall impedance is dominated by the solution resistance. At a low detection frequency, the interface impedance value of the plate electrode has the greatest impact on the impedance. Improper detection frequency selection would lead to a drop in detection sensitivity. Only within a certain frequency range, the impedance changes of the cell itself can be represented as much as possible. Due to the addition of the photoassisted effect, the experimental duration was within 2 h, and the influence of the culture medium alone could be ignored. The change in solution impedance after adding the drug is the key proof of the effectiveness of the experiment. The relevant pre‐experiment results are shown in Figure S1 (Supporting Information).

### Establishment of an Impedance Model for Cell State Assessment

The cell impedance values under different concentrations of H_2_O_2_ and different doses of UVA exposure in the preliminary experiment, indicating that the static impedance reflects the activity strength of cells under specific stress, were measured. In order to relate the cell impedance value to the cell state, the cell viability (*CV*) was defined as the ratio of the zero‐impedance value:

(6) $$CV=\frac{Z_{P}-Z_{0}}{Z_{100}-Z_{0}}\times 100\%$$

where *Z*_p_ is the impedance value of cells under different stresses at zero irradiation, *Z*_100_ is the impedance value of cells without stress, and *Z*_0_ is the impedance value of completely dead cells.

The cell resistibility (*CR*) can be reflected in the descent rate of cell impedance under UVA exposure, *k*,

(7) $$k=\frac{\Delta Z}{60}$$

where Δ*Z* is the difference in impedance before and after light stimulation. The higher the *k* value is, the lower the *CR* is. If the *CR* of the untreated cells is defined as 100%, and the *CR* at the maximum impedance descent rate is defined as 0%, then the *CR* in ECIS measurement can be normalized as

(8) $$CR\left(\%\right)=\frac{k_{P}-k_{0}}{k_{100}-k_{0}}\times 100\%$$

where *k*_p_ is the descent rate of cell impedance under light stimulation, *k*_100_ is the descent rate of cell impedance of untreated cells, and *k*_0_ is the descent rate of cell impedance when the cells are barely alive. When reached *k*_0_, even if the cells were partially alive, they completely lost their ability to resist external stimuli.

## Conflict of Interest

The authors declare no conflict of interest.

## Supporting information



Supporting Information

## Data Availability

The data that support the findings of this study are available from the corresponding author upon reasonable request.
