# Supplementary material for: Cell Viability, Drug Screening, and Mechanism Study Under Mild Phototherapy Integrated Chemotherapy (PIC)
Source: Adv Sci (Weinh). 2025 Aug 29;12(45):e02836. doi: 10.1002/advs.202502836 (PMC12677658; doi:10.1002/advs.202502836)
Supplement: Supplementary file 1 — Supporting Information [file ADVS-12-e02836-s001.docx]

**Attachment**


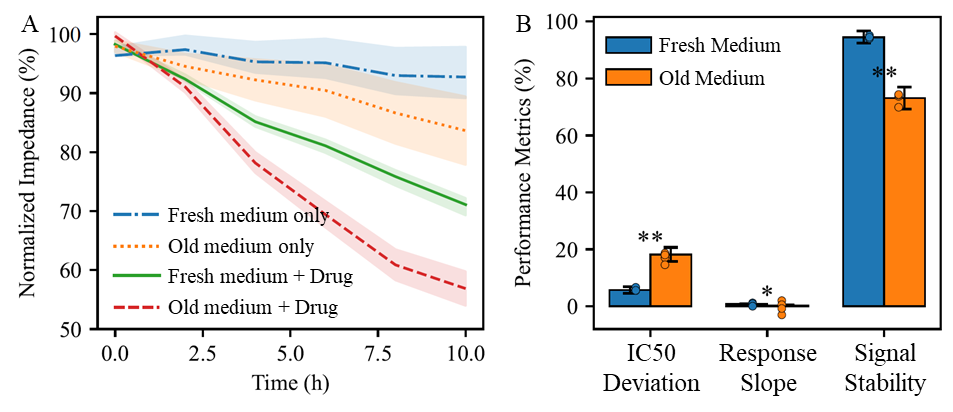


**Fig.S1.** The influence of culture medium on the pre-experiment **(A)** Time-varying dynamic characteristics of impedance under different culture conditions; **(B)** Comparison of the influence of culture conditions on multi-parameter detection performance


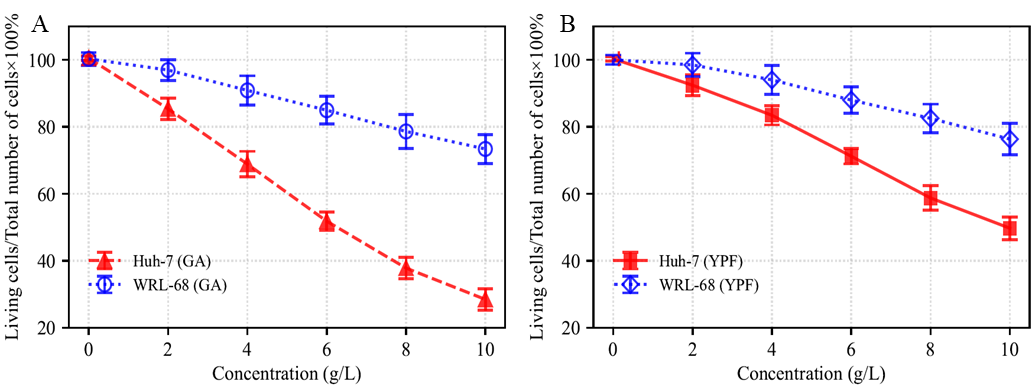


**Fig.S2.** **The effects of different drug doses on cells (A)** GA Dose Response; **(B)** YPF Dose Response.
